# COVID‐19, *dugnad* and productive incompleteness: volunteer labour and crisis loans in Norway

**DOI:** 10.1111/1469-8676.12814

**Published:** 2020-05-13

**Authors:** Knut Christian Myhre

**Affiliations:** ^1^ Museum of Cultural History University of Oslo Oslo 0164 Norway

Starting on 12 March, the Norwegian government decreed a range of measures in response to the COVID‐19 pandemic. Like similar efforts elsewhere, their objective was to provide healthcare for those infected and slow the spread of the virus among the population. Yet, these infection control measures proved at least as effective at slowing down economic activity. They therefore necessitated further interventions to provide liquidity and support for corporations and households facing a sudden loss of income. Many of the measures are familiar from the 2008 financial crisis and differ only in the speed with which they were deployed in the current situation.

It is interesting to note how all actors from the start described these measures as a national *dugnad*. *Dugnad* is a form of unpaid voluntary labour that is common in non‐commercial groups and organisations in Norway. Thus, housing co‐op inhabitants join in *dugnad* every spring and spend an afternoon cleaning, gardening and performing maintenance. Similarly, parents organise flea‐markets on *dugnad* to fund the marching bands at their children’s schools, and sell hot‐dogs and waffles during game days for the benefit of local sports clubs. We often complain about *dugnad* as an inconvenient imposition, but also acknowledge it as a means to complete tasks for which we would otherwise need to pay and a way to get to know our neighbours. In short, *dugnad* is both a pragmatic mode of organisation and a social value. Accordingly, the noun *dugnad* with its Old Norse origin relates to *dygd* that designates virtue or a valued capacity, and both derive from the verb *duge*, which means to be of use or avail.

Yet, there is more at stake in the *dugnad* responding to COVID‐19 than that individuals come together for the common good. More specifically, the economic interventions manifest what I call *productive incompleteness*, where an actor enrols and enables others to complete its efforts. It is perhaps most evident in the loan‐guarantee facility for the airline industry presented on 19 March. Here, the government offers guarantees for 90% of the value of loans totalling six billion Norwegian kroner, but only if a financial institution guarantees for the rest and sets the terms for the loans given. The state guarantee hence requires completion by a commercial actor, which in turn enrols other lenders to ensure that credit extends on market terms. It goes even further for the financially stressed low‐cost carrier Norwegian, which only gets access to the full guarantee if existing creditors waive interest and postpone repayments, and shareholders increase equity. Unlike 2008, this is not a government bailout that socialises losses and privatises gains. Instead, it is a *dugnad* where the state enrols different financial actors that contribute in different ways to complete an arrangement to ensure the continued existence of a company deemed part of vital infrastructure. As such, it show the appeal to and of a notion with a long history and intimate involvement in people’s lives for responding to an unprecedented situation in ways that avoid the pitfalls of a crisis past.

